# Mycoplasma pneumoniae-Triggered Guillain-Barré Syndrome in Children: Two Case Reports of Different ICU Presentations

**DOI:** 10.7759/cureus.74492

**Published:** 2024-11-26

**Authors:** Sajjad M AlKadhem, Alzahra Alradhi, Hadeel A AlJubab, Ali H AlWadei

**Affiliations:** 1 Pediatrics Intensive Care, King Fahad Medical City, Riyadh, SAU; 2 Pediatrics, King Fahad Medical City, Riyadh, SAU; 3 Pediatric Intensive Care, King Fahad Medical City, Riyadh, SAU; 4 Pediatric Neurology, King Fahad Medical City, Riyadh, SAU

**Keywords:** guillain-barré syndrome, mycoplasma pneumoniae, pediatric intensive care, pharyngeal-cervical-brachial variant, sickle cell anemia

## Abstract

Guillain-Barré syndrome (GBS) is an acute immune-mediated polyneuropathy with diverse clinical presentations. Mycoplasma pneumoniae has been increasingly recognized as a potential trigger, particularly in pediatric cases. This case report presents two atypical cases of M. pneumoniae-associated GBS in children in the pediatric intensive care unit setting. The first case involves a 10-year-old boy with pharyngeal-cervical-brachial variant of GBS, presented with descending paralysis and required prolonged mechanical ventilation and tracheostomy. The second case presents a two-year-old boy with ascending GBS complicated by underlying sickle cell anemia.

Both cases illustrate that pediatric GBS requires prompt diagnosis and aggressive treatment. In these instances, GBS was linked to M. pneumoniae.

## Introduction

Guillain-Barré syndrome (GBS) remains the most common cause of acute flaccid paralysis worldwide, with a reported incidence of 0.81 to 1.89 variant forms exist, including the pharyngeal-cervical-brachial (PCB) variant, which accounts for approximately 3%-5% of GBS cases [2].

Mycoplasma pneumoniae has been increasingly recognized as a potential trigger for GBS, with research suggesting it may be responsible for up to 15%-20% of pediatric GBS cases [3]. The management of GBS often requires intensive care, with a significant proportion of pediatric patients requiring mechanical ventilation [4]. This report presents two atypical cases of M. pneumoniae-associated GBS, emphasizing the diagnostic challenges, intensive care unit management strategies, and outcomes in pediatric patients.

## Case presentation

Case 1: pharyngeal-cervical-brachial variant GBS

A previously healthy 10-year-old boy presented with a one-week history of upper respiratory tract infection symptoms, including low-grade fever, cough, and severe dysphagia. Initially treated with antibiotics for suspected pneumonia, the patient's condition worsened, developing severe upper limb weakness. Upon admission to the pediatric intensive care unit (PICU), he exhibited bilateral ptosis, facial diplegia, and absent deep tendon reflexes.

**Figure 1:**
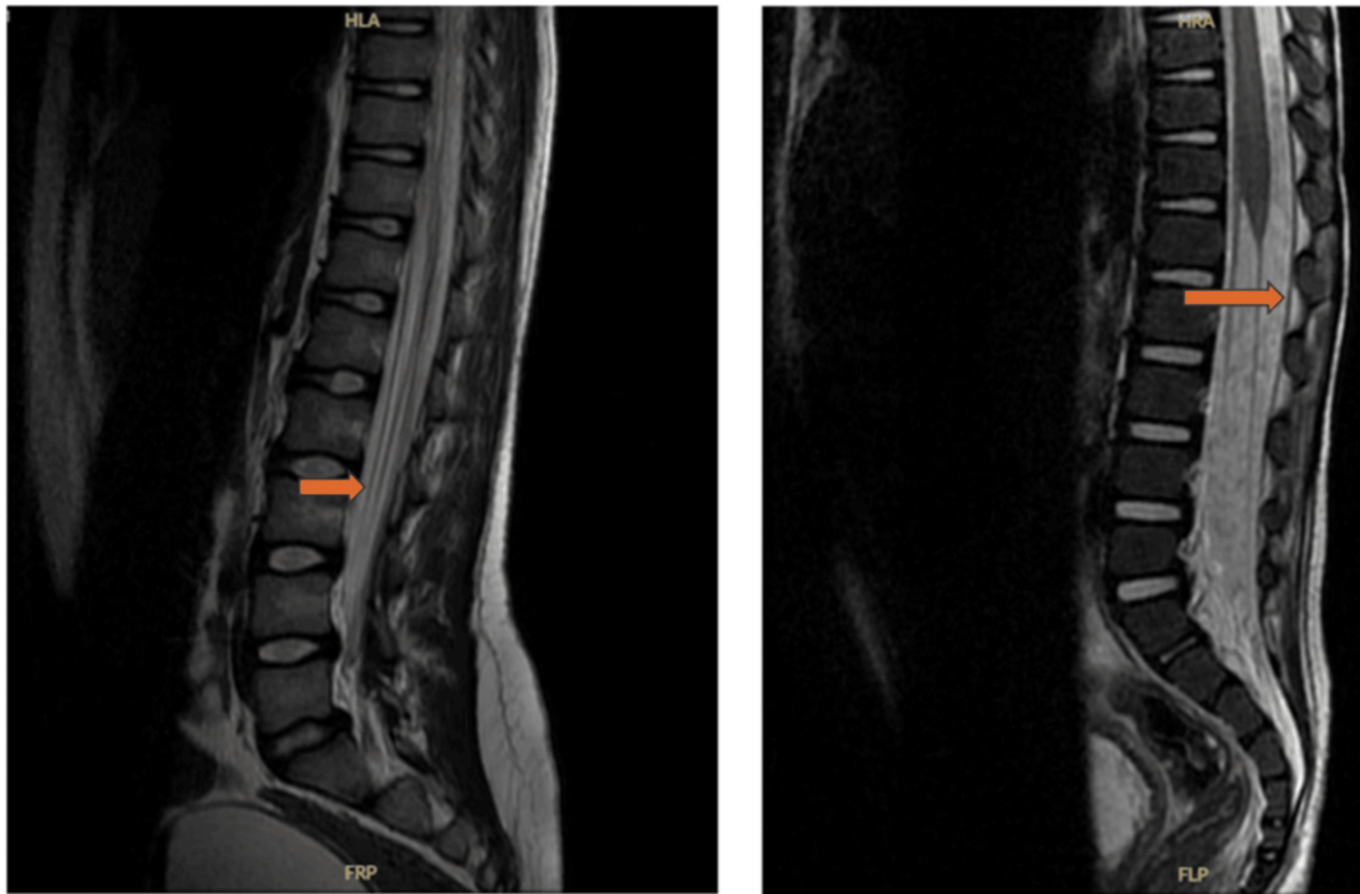
Abnormal spinal cord signal intensity at the lower thoracic and conus medullaris level, with enhancement of the cauda equina nerve roots.

**Figure 2:**
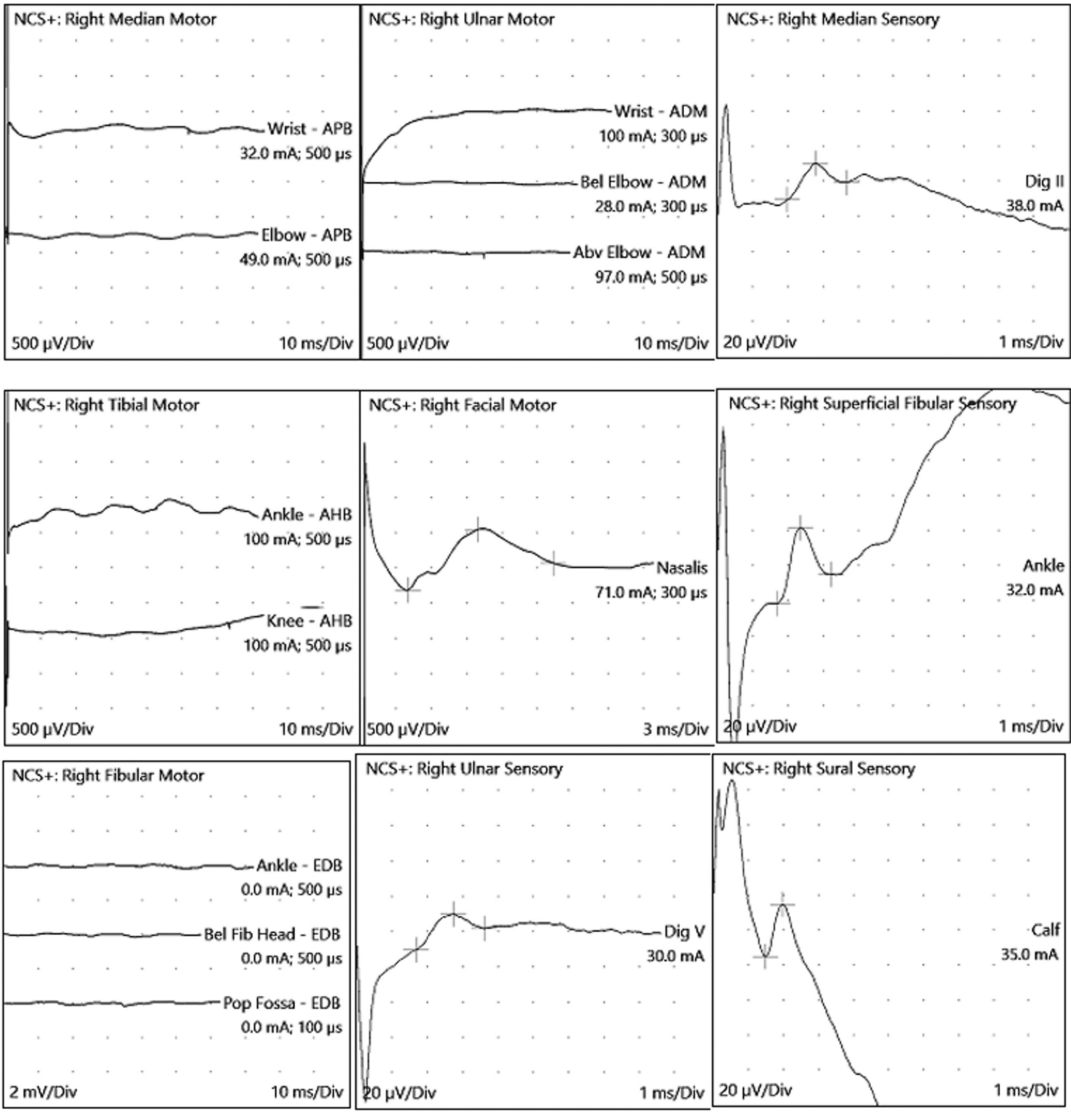
Nerve conduction studies waveforms (Case 1).

The patient was intubated due to progressive descending paralysis affecting respiratory effort. Magnetic resonance imaging (MRI) of the brain and spine showed abnormal spinal cord signal intensity at the lower thoracic and conus medullaris level, with enhancement of the cauda equina nerve roots (Figure 1). Serial nerve conduction studies demonstrated progression from predominantly motor axonal polyneuropathy to mixed sensorimotor axonal polyneuropathy (Table 1, Figure 2).

**Table 1:** Results of nerve conduction studies (Case 1 and Case 2) NCS: nerve conduction studies; EDB: extensor digitorum brevis; APB: abductor pollicis brevis; AHB: abductor hallucis muscle; ADM: abductor digiti minimi; NR: not recordable; ms: millisecond; mV: millivolt, m/s: meter per second.

NCS parameters		Case 1	Case 2	Normal value	NCS parameters	Case 1	Case 2	Normal value
		Right motor	Left motor			Right Sensory (Start – Peak)	Left Sensory (Start – Peak)	
Latency (ms)	Fibular EDB	NR	NR	≤6.1	Superficial fibular	NR	NR	≤4.1
	Median APB	NR	NR	≤4.2	Median	NR	1.35 – 1.95	≤3.6
	Tibial AHB	NR	NR	≤6.1	Sural	1.00 – 1.50	1.25 – 1.65	≤4
	Ulnar ADM	NR	NR	≤4.2	Ulnar	NR	1.28 – 1.93	≤3.7
Amplitude (mV)	Fibular EDB	NR	NR	≥2	Superficial fibular	NR	NR	≥5
	Median APB	NR	NR	≥5	Median	NR	22 – 28	≥10
	Tibial AHB	NR	NR	≥4.4	Sural	10 – 15	3 – 4	≥5
	Ulnar ADM	NR	NR	≥3	Ulnar	NR	16 – 14	≥15
Velocity (m/s)	Fibular EDB	NR	NR	≥38	Superficial fibular	NR	NR	≥32
	Median APB	NR	-	≥50	Median	NR	59	≥39
	Tibial AHB	NR	-	≥39	Sural	60 – 40	56	≥35
	Ulnar ADM	NR	-	≥53	Ulnar	NR	55	≥38

Cerebrospinal fluid (CSF) analysis, with normal white blood cell count, protein, and glucose levels. Polymerase chain reaction (PCR) testing was positive for M. pneumoniae.

Treatment was initiated with intravenous immunoglobulin (IVIG) at 2g/kg divided over two days, he was showing no significant improvement so was followed by five sessions of plasma exchange (PLEX) after one week. On day 20, a tracheostomy was performed to facilitate long-term airway management. After 45 days in the PICU, the patient was transitioned to an aerosol tracheostomy mask, showing gradual improvement in motor function.

Case 2: ascending GBS in a child with sickle cell anemia

A two-year-old boy with known sickle cell anemia presented with lower back pain and fever. Initially treated for a vaso-occlusive crisis, the patient developed ascending weakness 10 days later, which rapidly progressed to respiratory failure requiring PICU admission and intubation.

**Figure 3:**
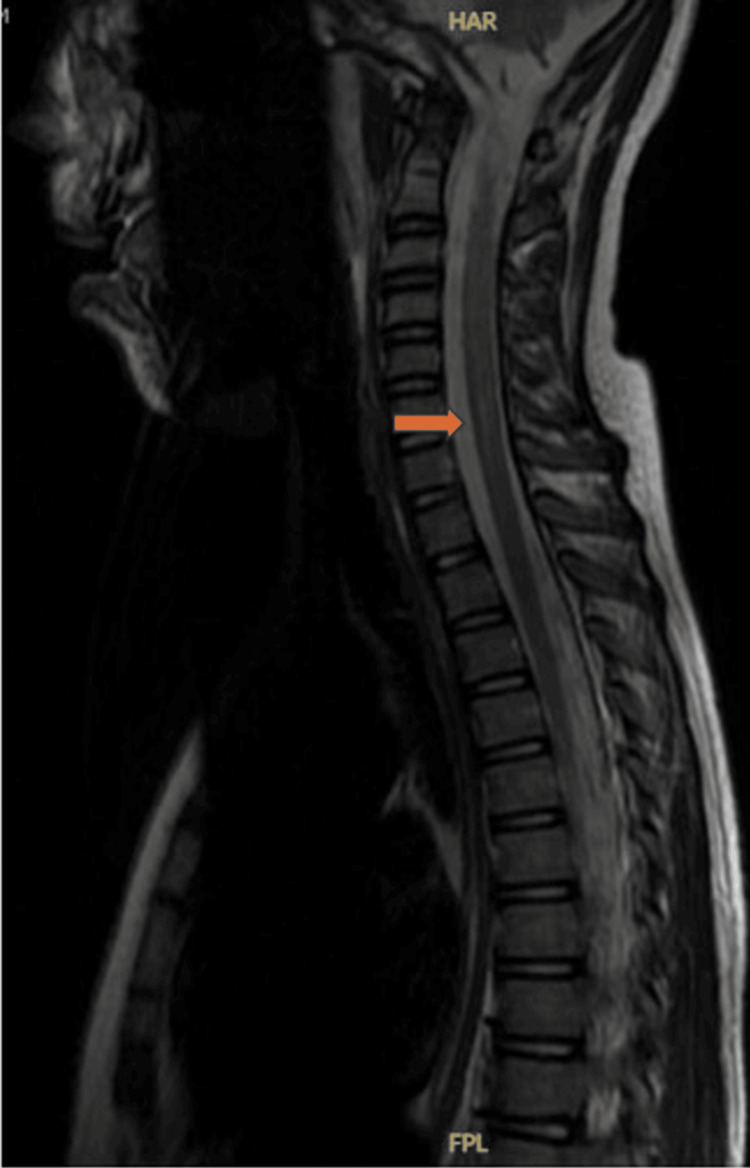
MRI of the spine showing abnormal enhancement of ventral and dorsal nerve roots of the cauda equina.

**Figure 4:**
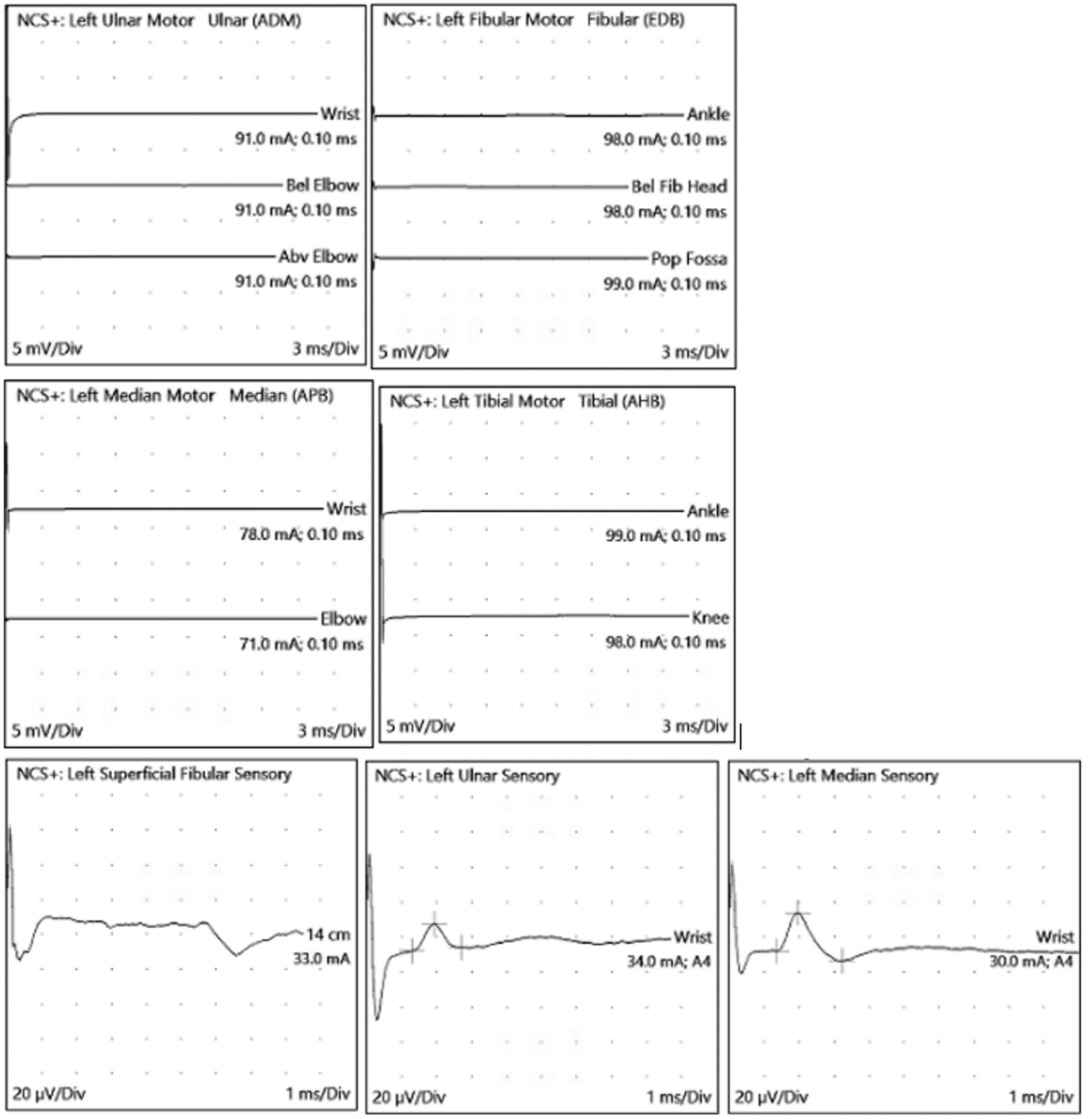
Nerve conduction studies waveforms (Case 2).

CSF analysis revealed elevated protein levels, demonstrating the classic albuminocytological dissociation. MRI of the spine showed abnormal enhancement of ventral and dorsal nerve roots of the cauda equina (Figure 3). Nerve conduction studies suggested a sensorimotor (predominantly motor) axonal polyneuropathy (Table 1, Figure 4). PCR testing was positive for M. pneumoniae.

Treatment was initiated with IVIG at 400 mg/kg/day for five days, followed by seven sessions of PLEX. After 18 days of mechanical ventilation, the patient was successfully extubated to high-flow nasal cannula oxygen therapy. He was transitioned to room air after 28 days in the PICU and subsequently transferred to the ward for continued rehabilitation, showing significant improvement in motor function.

## Discussion

These two cases highlight the diverse presentations of GBS in pediatric patients and underscore the importance of considering this diagnosis even in atypical scenarios, particularly in the intensive care setting. As seen in Case 1, the PCB variant is characterized by rapidly progressive oropharyngeal and cervicobrachial weakness associated with areflexia in the upper limbs [2]. This variant can be challenging to diagnose due to its localized nature and potential mimics such as botulism or myasthenia gravis [5].

The association of GBS with M. pneumoniae infection, observed in both cases, is a crucial finding. Recent studies have further solidified M. pneumoniae as a significant trigger for GBS, particularly in pediatric populations [3]. This underscores the importance of testing for M. pneumoniae in patients presenting with symptoms suggestive of GBS, even in the absence of typical respiratory symptoms.

A case series published in 2015 highlights the association between M. pneumoniae infection and severe GBS [6]. In this series, 47% of patients with severe GBS were found to have Mycoplasma pneumonia. Most patients were previously healthy and exhibited respiratory and gastrointestinal prodromal symptoms preceding the onset of neurological manifestations. All reported cases with CSF analysis revealed elevated protein levels, contrasting with our first case. Notably, no reported cases of the PCB variant of GBS were associated with M. pneumoniae. Furthermore, all cases were classified within the acute inflammatory demyelinating polyneuropathy type, as suggested by the nerve conduction study, whereas our cases showed predominantly motor axonal polyneuropathy [6].

Case 2 presents a more typical ascending pattern of GBS but is noteworthy due to the patient's underlying sickle cell anemia. The co-occurrence of GBS and sickle cell disease is rare, with few documented cases highlighting potential challenges in diagnosis and management [7]. This case emphasizes the need for vigilance in recognizing GBS in patients with pre-existing hematological conditions that might complicate the clinical picture and ICU management.

Both cases demonstrate the utility of combining clinical, electrophysiological, and neuroimaging findings in diagnosing GBS in the ICU setting. Recent guidelines emphasize the importance of this multimodal approach to diagnosis, particularly in atypical presentations [8]. The absence of albuminocytological dissociation in the CSF of Case 1 highlights that normal CSF protein does not exclude GBS, especially early in the disease course [9].

The ICU management of these cases followed current guidelines for severe GBS, utilizing IVIG and PLEX. The use of PLEX after initial IVIG treatment in both cases aligns with emerging evidence suggesting that some patients may benefit from this sequential approach, particularly in severe or refractory cases [10].

## Conclusions

These cases illustrate the importance of recognizing atypical presentations of GBS in pediatric patients, particularly in the context of M. pneumoniae infection. Clinicians should maintain a high index of suspicion for GBS variants and consider the diagnosis even in the presence of confounding factors such as pre-existing conditions or atypical CSF findings. A comprehensive infectious workup is essential in suspected cases. Diagnosis and treatment should involve electrophysiological studies, neuroimaging, and immunotherapy. The ICU management of pediatric GBS requires a delicate balance of respiratory support and immunomodulatory therapy while monitoring for complications. Early intervention is vital to avoid potential prolonged ICU stays and rehabilitation needs. Future research should optimize ICU strategies and explore M. pneumoniae role in GBS.
